# Targeting Lipophagy in Liver Diseases: Impact on Oxidative Stress and Steatohepatitis

**DOI:** 10.3390/antiox14080908

**Published:** 2025-07-24

**Authors:** Jin Seok Hwang, Trang Huyen Lai, Deok Ryong Kim

**Affiliations:** Department of Biochemistry and Convergence Medical Sciences and Institute of Medical Science, College of Medicine, Gyeongsang National University, Jinju 52727, Republic of Korea; jsh901231@gnu.ac.kr (J.S.H.); tranghuyen@gnu.ac.kr (T.H.L.)

**Keywords:** lipophagy, MASLD, MASH, oxidative stress, hepatic steatosis, lipotoxicity, metabolic dysfunction

## Abstract

Metabolic dysfunction-associated steatotic liver disease (MASLD) encompasses a range of liver conditions, from simple hepatic steatosis to its more severe inflammatory form known as metabolic dysfunction-associated steatohepatitis (MASH). Despite its growing clinical significance and association with cirrhosis and cancer, there are currently few pharmacological treatments available for MASLD, highlighting the urgent need for new therapeutic strategies. This narrative review aims to elucidate the molecular mechanisms of lipophagy in MASLD progression, emphasizing how its dysfunction contributes to hepatic steatosis and lipotoxicity. We also explore the intersection of lipophagy failure with oxidative stress and inflammation in the liver, focusing on key signaling pathways, such as mTORC1 and AMPK, and discuss the therapeutic potential of targeting these pathways by systematically reviewing the literature from PubMed, Scopus, and Google Scholar databases. Recent studies suggest that lipophagy, the selective autophagic degradation of lipid droplets, is crucial for maintaining hepatic lipid homeostasis. Indeed, some vital components of the lipophagy machinery seem to be functionally inhibited in MASLD, resulting in the accumulation of intracellular triacylglycerol (TAG), lipotoxicity, and subsequent oxidative stress, all of which contribute to disease progression. In summary, impaired lipophagy is a central pathological mechanism in MASLD, making it an important therapeutic target. A deeper understanding of these mechanisms may offer new strategic insights for combating the progression of MASLD/MASH.

## 1. Introduction

Metabolic dysfunction-associated steatotic liver disease is recognized as the most prevalent chronic liver condition globally, impacting over 30% of the world’s population [1]. Previously known as NAFLD (non-alcoholic fatty liver disease), the former term highlighted the exclusion of significant alcohol consumption as a defining characteristic [2,3]. However, the old term did not adequately represent the strong link between the condition and underlying metabolic dysfunctions. In 2023, a global initiative adopted the term MASLD to highlight the central role of metabolic dysfunction in its pathogenesis and to enhance diagnostic and therapeutic approaches [4]. MASLD encompasses a range of liver disorders marked by hepatic steatosis and associated metabolic risk factors, such as obesity, type 2 diabetes, and dyslipidemia [5]. A particularly concerning subset of MASLD is metabolic dysfunction-associated steatohepatitis (MASH), mainly when fibrosis occurs, which can progress to cirrhosis, liver failure, and hepatocellular carcinoma [6]. The severity of fibrosis is the most significant predictor of liver-related and overall mortality. The transition from hepatic steatosis to MASH and advanced fibrosis involves a complex interaction of pathogenic factors, including insulin resistance and the buildup of harmful lipid intermediates that cause hepatocellular damage and fibrogenesis [7]. Oxidative stress and mitochondrial dysfunction further aggravate this progression, fostering inflammation and hepatocellular damage crucial to MASH development [8,9,10].

A key early feature of MASLD is the excessive buildup of lipid droplets (LDs) within hepatocytes [11]. Surplus fatty acids are converted into triacylglycerols (TAGs) and stored within cytoplasmic LDs in nutrient-rich conditions. These dynamic organelles are vital for lipid storage, trafficking, and metabolic signaling [12,13]. Structurally, LDs comprise a neutral lipid core, mainly TAGs and cholesterol esters, encased in a phospholipid monolayer embedded with various proteins crucial for lipid storage and metabolism [14]. Lipid droplet proteins, especially those from the perilipin (PLIN) family on the LD surface, play specific roles in lipid storage and mobilization under different metabolic conditions [15]. Proper LD formation and turnover regulation are critical for maintaining hepatic lipid balance. Disruption of this balance, whether through excessive lipid accumulation, impaired disposal via β-oxidation, or very low-density lipoprotein (VLDL) secretion, leads to hepatic steatosis, lipotoxicity, and the progression of MASLD [16,17].

Lipophagy, the selective autophagic degradation of lipid droplets, is a crucial cellular process for managing lipid overload in the liver [18]. This catabolic pathway is essential for mobilizing stored lipids to provide energy and acts as a primary defense mechanism against lipid-induced cellular stress, thereby supporting metabolic and redox balance [19,20]. Recent evidence indicates that impaired lipophagy may contribute to the development of MASLD [21,22]. However, a comprehensive understanding of how lipophagy impairment leads to lipotoxicity, oxidative stress, and sterile inflammation is still lacking. This knowledge gap is critical in light of the recent redefinition of MASLD, underscoring the pathophysiological importance of lipophagy. This review seeks to address this gap by proposing that lipophagy impairment is not only a result of steatosis, but also a key driver that triggers and perpetuates the lipotoxic and inflammatory processes characteristic of MASLD. To support this hypothesis, the review explores the molecular mechanisms that regulate lipophagy, its dysregulation in MASLD, and the resulting pathological outcomes. It will emphasize how disruptions in lipophagy contribute to oxidative stress, inflammation, and the progression of liver disease. Additionally, the review discusses the potential therapeutic benefits of targeting this pathway, especially in maintaining oxidative balance. For the scientific community, this review offers an integrated pathological model that unifies various molecular findings into a coherent framework, which can guide future research and aid in identifying new therapeutic targets.

## 2. Molecular Mechanisms of Lipophagy

### 2.1. Core Autophagy Machinery to Target Lipid Droplets

**Figure 1:**
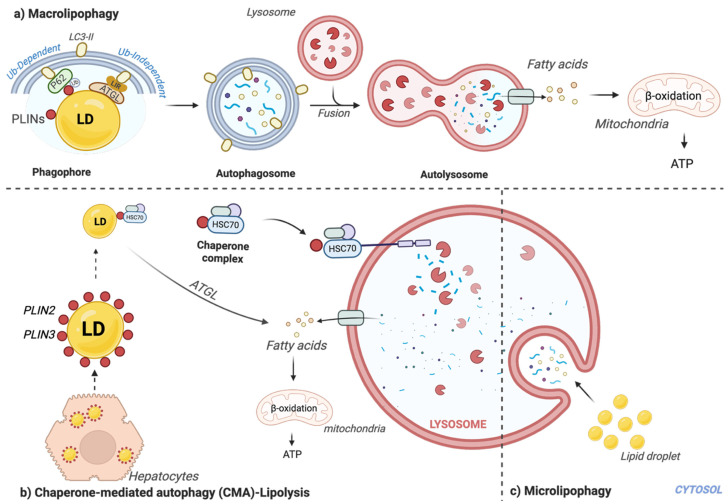
A schematic overview illustrates the lipophagy-dependent degradation of lipid droplets in hepatocytes. (**a**) Macrolipophagy: Lipid droplets are encased by a double-membraned structure known as a phagophore, which is positive for LC3-II. This encapsulation process involves two main pathways, as follows: ubiquitin-dependent and ubiquitin-independent macrolipophagy. In the ubiquitin-dependent pathway, specific lipid droplet coat proteins, such as Perilipin 1 (PLIN1) and Perilipin 2 (PLIN2), are tagged with poly-ubiquitin chains. These tags allow for recognition by p62, which then facilitates macroautophagy. Conversely, in the ubiquitin-independent pathway, lipid droplet-associated proteins like adipose triglyceride lipase (ATGL) bind directly to LC3-II through their LC3-interacting region (LIR) motifs, initiating autophagic degradation without the need for ubiquitination. Once these pathways capture lipid droplets, the phagophore expands and closes to form a complete autophagosome. This vesicle fuses with a lysosome to create an autolysosome, where lysosomal enzymes break down the engulfed lipid droplets, releasing their contents. The liberated fatty acids are then transported to the mitochondria for β-oxidation, generating ATP for cellular energy. (**b**) Chaperone-mediated autophagy (CMA): Lipid droplets, coated with proteins such as PLIN2 and PLIN3, are recognized by a chaperone complex that includes HSC70. This complex guides the PLINs to the lysosome for degradation by CMA. LDs without PLIN coats can be degraded by macro- or microlipophagy, or release fatty acids by the action of adipose triglyceride lipase (ATGL), used for mitochondrial β-oxidation and ATP generation. (**c**) Microlipophagy: Lipid droplets are directly engulfed by the lysosome and undergo degradation through phagocytosis. LD: Lipid droplet; Ub: Ubiquitin; LIR: LC3-interacting region. The illustration was mainly supported by these references [28,30,33,38,46,50].

Lipophagy, the selective autophagic degradation of LDs, operates through the following three primary pathways: macroautophagy/macrolipophagy, chaperone-mediated autophagy (CMA), and microautophagy/microlipophagy (Figure 1). The activation of these pathways can differ based on cell type, metabolic conditions, and the characteristics of the LDs.

Macrolipophagy is a classical pathway where LDs are degraded through autophagosome-mediated processes. This mechanism involves a double-membraned vesicle known as an autophagosome, which engulfs an entire LD and transports it to the lysosome for degradation. The process depends on several autophagy-related proteins as molecular links between LDs and the core autophagic machinery. LDs are recruited to the autophagosome via the following two primary mechanisms: ubiquitin-dependent and ubiquitin-independent pathways. In the ubiquitin-dependent pathway, ubiquitin (Ub), a small protein tag, marks LDs for degradation [23]. Initially, proteins on the LD surface are tagged with ubiquitin chains, signaling them for degradation [24]. Spartin/SPG20 is a crucial protein facilitating this ubiquitination process [25,26]. Two key LD surface proteins, Perilipin 1 (PLIN1) and Perilipin 2 (PLIN2), are involved in this pathway. They have been shown to coimmunoprecipitate with p62 [27,28] and are degraded through the ubiquitination–proteasome pathway [24,29]. Adaptor proteins like p62/SQSTM1 and NBR1 link ubiquitinated LDs to the autophagic machinery by interacting with lipidated LC3/GABARAP proteins (LC3-II) [30]. Proteins like huntingtin (HTT) further support this process by acting as scaffolds that assist p62 in recognizing and binding both ubiquitinated proteins and LC3-II [31]. Core autophagy proteins such as ATG14 and WIPI proteins initiate the process by localizing near the LD surface [32,33]. Conversely, ubiquitin-independent macrolipophagy involves direct interactions between LD-associated proteins and the autophagosome. This mechanism relies on proteins containing an LC3-interacting region (LIR) motif, which allows them to directly bind to LC3-II on the autophagosome membrane. Key cytosolic lipases like adipose triglyceride lipase (ATGL) and hormone-sensitive lipase (HSL) contain the LIR motif, facilitating the recognition and encapsulation of LDs into the forming autophagic vesicle [34]. A recent study identified TP53INP2, a transcription factor regulating autophagy, as a potential adaptor protein that connects LDs with lipophagy cargos. Coimmunoprecipitation analysis confirmed the direct interaction of TP53INP2 with PLIN1 and the autophagosome receptor LC3-II through its LIR motif, particularly in the absence of p62 in the complex [35]. Once LDs are sequestered by either mechanism, the autophagosome membrane must extend and close around them. This process is driven by the following two critical ubiquitin-like conjugation systems: the ATG12-ATG5-ATG16L1 complex and the conjugation of LC3 to the membrane lipid PE, forming LC3-II [36]. The final coating of LC3-II on the completed autophagosome is essential for its maturation and successful fusion with the lysosome, where the lipid droplet is ultimately degraded.

Chaperone-mediated autophagy (CMA) is another highly selective lipophagy targeting specific LD-associated proteins for lysosomal degradation. This process begins when the cytosolic chaperone HSPA8/HSC70 recognizes proteins with a KFERQ-like motif. Key LD surface proteins, such as Perilipin 2 (PLIN2) and Perilipin 3 (PLIN3), have been identified as CMA substrates [37,38]. Rab GTPases (RAB7, RAB10, RAB32) may facilitate these interactions [39,40,41]. The HSPA8/HSC70/PLIN complex docks at the lysosomal membrane via interaction with LAMP2A, the CMA receptor [38,42]. Following binding, LAMP2A multimerizes, and the substrate protein is unfolded and translocated into the lysosomal lumen for degradation. The removal of PLIN2 and PLIN3 destabilizes the LD surface, exposes neutral lipids, and makes them more accessible to cytosolic lipases like ATGL for lipolysis or macrolipophagy engulfment, allowing for the breakdown of TAGs and the release of fatty acids for energy [43,44,45].

Microlipophagy involves directly engulfing LDs or portions of them by the lysosomal or vacuolar membrane, without forming a separate autophagosome [46]. This process, distinct from macrolipophagy, has been extensively studied in yeast (e.g., *Saccharomyces cerevisiae*) [47]. Microlipophagy can be categorized into the following three subtypes based on membrane dynamics: lysosomal protrusion, lysosomal invagination, and endosomal invagination. It is driven by core ATG proteins such as ATG7, ATG8, ATG14, and VPS30/ATG6, and components of the endosomal sorting complexes required for transport (ESCRT) machinery, including VPS27 [48]. While primarily characterized in yeast, some evidence suggests that microlipophagy occurs in mammalian hepatocytes. A study by Schulze et al. in 2020 reported microlipophagy induction in hepatocytes, independent of macrolipophagy or CMA [49]. Increasing the size of LDs in vitro inhibits lipophagy, indicating that large LDs are more challenging for lysosomes to process [50,51]. Another study reported that LD–lysosome trafficking in hepatocellular lipophagy depends on RAB5 GTPase [52]. However, the precise molecular mechanisms and physiological significance of microlipophagy in mammals remain to be fully understood.

Despite their distinct mechanisms, all lipophagy pathways converge on the lysosomal degradation of LDs, resulting in the hydrolysis of TAGs and cholesterol esters into free fatty acids and cholesterol. These molecules are then available for various cellular functions, including energy production, membrane synthesis, and other metabolic processes [37].

### 2.2. Key Regulatory Pathways

Lipophagy is particularly regulated by a complex network that integrates signals related to cellular nutrient status, metabolic cues, and hormonal influences. This intricate regulation ensures the appropriate mobilization of lipid stores, maintaining cellular homeostasis. Central to this regulation are critical nutrient-sensing signaling pathways. Key among these are the AMP-activated protein kinase (AMPK) and the mechanistic target of Rapamycin complex 1 (mTORC1), which act as primary regulators of cellular metabolism. In nutrient-rich conditions, mTORC1 is activated, suppressing autophagy initiation by inhibiting the ULK1 complex, an essential initiator of autophagosome formation [53]. Conversely, during nutrient scarcity or energy stress, AMPK is activated. AMPK not only inhibits mTORC1 but also directly phosphorylates and activates ULK1, thereby promoting autophagic processes, including lipophagy [54].

Transcriptional regulation adds another layer of control. Transcription factors, such as TFEB and TFE3, master regulators of lysosomal biogenesis and autophagy, and enhance lipophagy by upregulating key *Atg* and lysosome-related genes [55,56]. When mTORC1 is active, it phosphorylates TFEB and TFE3, causing them to remain in the cytoplasm. However, when mTORC1 is inhibited (such as during starvation), dephosphorylated TFEB and TFE3 move to the nucleus, stimulating the expression of a wide array of *Atg* and lysosomal genes, thereby significantly promoting lipophagy [56].

Systemic hormonal control also modulates lipophagy. For instance, insulin inhibits autophagy by activating mTORC1, while glucagon induces autophagy during fasting, reflecting the liver’s response to systemic energy signals [57]. Additionally, regulation occurs at the lipid droplet surface itself. Specific lipid droplet surface proteins, particularly members of the PLIN family (e.g., PLIN2, PLIN3, PLIN5), play a regulatory role by either facilitating or obstructing the access of autophagic machinery to lipid droplets [58,59,60].

### 2.3. Interaction with Other Lipid Metabolic Pathways

Lipophagy is a comprehensive process that interacts with other critical lipid metabolic pathways, such as cytosolic lipolysis, mitochondrial β-oxidation, and VLDL secretion. The interplay among these pathways is vital for maintaining liver homeostasis, and disruptions in this balance are a hallmark of MASLD.

Beyond the direct mechanisms of engulfment, lipophagy is closely linked with cytosolic lipolysis, another primary pathway for mobilizing lipids from LDs. This pathway is mediated by essential lipases, primarily adipose triglyceride lipase and hormone-sensitive lipase, and the interaction between these systems is crucial for liver homeostasis [61]. Their relationship is both cooperative and compensatory; for example, CMA can target the LD coat protein PLIN2 for degradation. Removing PLIN2 destabilizes the LD surface, making the triacylglycerol core more accessible to both the macrolipophagy machinery and cytosolic lipases, demonstrating a synergistic interaction [38]. During nutrient deprivation, lipophagy and lipolysis are co-upregulated at the transcriptional level by master regulators like TFEB to ensure a coordinated release of FFAs for energy [62,63]. However, these pathways can also compensate for each other. Mouse models with a genetic deficiency in lipophagy develop a relatively moderate fatty liver compared to mice lacking the primary cytosolic lipase, ATGL [64]. These pieces of evidence suggest that while cytosolic lipolysis partially compensates for the loss of lipophagy, the reverse is insufficient to prevent severe steatosis when the main lipolytic pathway is impaired.

The primary fate of FFAs released by lipophagy is mitochondrial β-oxidation for ATP production. In this scenario, lipophagy is the supplier and mitochondria is the consumer. Efficient coupling between these processes is crucial to maintain energy balance and prevent the toxic accumulation of FFAs [65,66]. However, this cooperative relationship breaks down when the rate of FFA released from lipophagy exceeds the mitochondrial oxidative capacity, leading to lipotoxicity. This imbalance results in incomplete fatty acid oxidation, generating toxic lipid intermediates and causing a surge in mitochondrial ROS production. This ROS, in turn, damages both the mitochondria and the autophagy machinery, establishing a destructive feedback loop of cellular injury [67,68].

In hepatocytes, TAGs can also be packaged into VLDL for export, a pathway that competes with lipophagy for the same TAG pool [69]. Beyond simple substrate competition, recent evidence reveals direct regulatory crosstalk between lipophagy and VLDL secretion. For instance, the signaling lipid sphingosine-1-phosphate (S1P) normally suppresses CMA to protect essential VLDL transport proteins, such as the SNAREs SEC22B, STX5A, and GS28, from degradation. In states of S1P deficiency, CMA can become overactive, destroying these proteins, halting VLDL secretion, and causing steatosis [70]. This mechanism highlights how specific regulatory imbalances, not just substrate availability, directly link lipophagy to the pathophysiology of MASLD. Therefore, lipophagy dysregulation does not occur in isolation, but triggers cascading failures in the interconnected pathways that govern hepatic lipid homeostasis, driving the progression of MASLD.

## 3. Lipophagy Dysregulation in MASLD

### 3.1. Impaired Lipophagy in MASLD

There is mounting evidence that lipophagy is compromised in metabolic dysfunction-associated steatotic liver disease [71]. Studies involving animal models and liver biopsies from patients with MASH consistently show disruptions in this crucial process [72,73,74]. A significant aspect of this impairment is the reduction in autophagic flux, indicating a decreased overall rate of autophagic degradation [75]. Interestingly, this can occur even with an increase in autophagosome numbers, suggesting a blockade in the later stages of degradation rather than a failure in their formation [71]. This impairment is further evidenced by the accumulation of autophagy markers like p62 [76]. Morphological studies also reveal that autophagosomes cannot effectively engulf lipid droplets or properly fuse with lysosomes [77].

Several interconnected mechanisms drive the impairment of lipophagy in MASLD. Chronic hyperinsulinemia and insulin resistance, core features of the disease, inhibit hepatic autophagy by causing persistent activation of the metabolic sensor mTORC1, a major inhibitor of autophagy initiation [78,79]. Concurrently, the inflammatory environment typical of MASH further hinders autophagic activity. For example, Toll-like receptor 4 (TLR4) signaling can upregulate Annexin A2 (ANXA2), which subsequently blocks autophagic flux mediated by the AMPK/mTOR pathway [80]. Additionally, lipotoxicity often leads to endoplasmic reticulum (ER) stress. While ER stress can initially trigger autophagy as a protective response, chronic ER stress impairs autophagic flux and exacerbates oxidative stress [81]. The chronic stress associated with MASLD also affects lysosomal acidification and reduces Cathepsin activity, disrupting lipophagy [75]. Indeed, a significant decrease in lysosomal enzyme activity, particularly lysosomal acid lipase (LAL), is often observed during the progression of MASLD, facilitating the impairment of lysosomal acidification [82,83]. Furthermore, another study found suppressed expression and activity of key lysosomal proteases, such as Cathepsins B, D, and L (CTSB, CTSD, CTSL), in the livers of patients with MASLD. This suppression contributes to the abnormality in lipophagic function observed in the disease [73].

Moreover, excessive oxidative stress poses a significant barrier to effective lipophagy. Unlike adaptive responses to moderate reactive oxygen species (ROS), the high levels typical in MASH conditions damage critical autophagy components (e.g., ATG4, lipids) and impair lysosomal function. This disruption creates a vicious cycle where defective autophagic clearance leads to ROS accumulation and cellular damage [84,85]. Finally, the sustained nutrient excess characteristic of MASLD reinforces mTORC1 activation, further suppressing lipophagy [86,87,88]. Despite growing evidence of impaired lipophagy in MASLD, important questions remain. It is still unclear whether these lipophagic defects are a cause or a consequence of the disease. Recent analyses underscore the need for further investigations to determine whether observed changes in autophagy, including lipophagy, represent an actual loss of function or a compensatory adaptation throughout different phases of MASLD progression [89].

### 3.2. The Dysfunction of Lipophagy in Steatosis and Lipotoxicity

**Figure 2:**
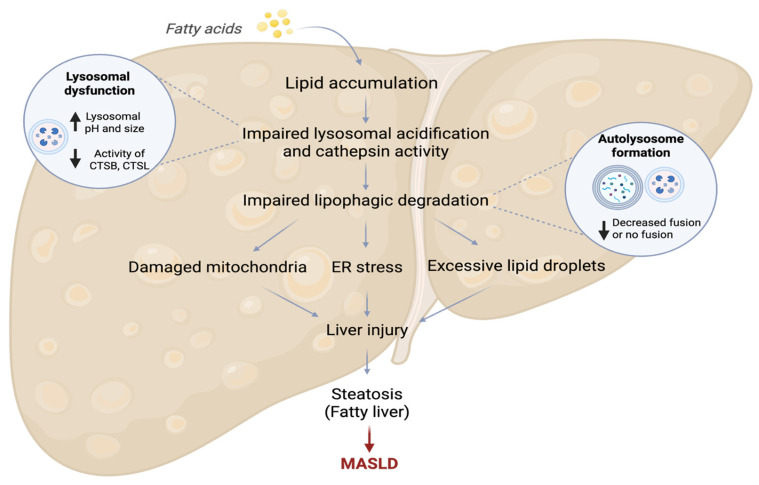
The dysregulation of lipophagy plays a significant role in the development and progression of MASLD. When lipid accumulation exceeds the hepatocyte storage capacity, lipophagic activity becomes impaired. Key lysosomal functions, such as acidification and Cathepsin-mediated degradation, are disrupted, leading to inefficient breakdown of lipid droplets. This dysfunction is often exacerbated by defective fusion between autophagosomes and lysosomes, limiting autolysosome formation and further compromising lipophagic degradation. Consequently, impaired lipophagy contributes to mitochondrial dysfunction, endoplasmic reticulum stress, and excessive intracellular lipid accumulation. This cascade of cellular damage promotes liver injury and steatosis, thereby accelerating MASLD progression. The illustration was mainly supported by these references [73,75,91].

The failure of lipophagy plays a crucial role in developing metabolic dysfunction-associated steatohepatitis (Figure 2). In the liver affected by MASLD, lipid accumulation exceeds the storage capacity of hepatocytes, leading to an increase in harmful lipid species such as saturated fatty acids, ceramides, and free cholesterol [90]. Importantly, this lipotoxic environment is not just a result of impaired lipid breakdown, but is also a primary factor contributing to the defect in lipophagy [91,92]. The impairment of lipophagy significantly hinders the degradation of lipid droplets in the liver, resulting in a substantial increase in triglyceride content within hepatocytes, which triggers steatosis [93]. The effects extend beyond simple fat storage, leading to stress on organelles, including mitochondrial damage and ER stress. These stresses, along with the direct impacts of lipotoxicity, converge to damage hepatocytes and promote inflammatory signaling [94]. As a result, impaired lipophagy contributes to fat accumulation and initiates key processes driving the progression of MASH [95,96]. These events are critical in advancing MASLD, underscoring the essential role of functional lipophagy in maintaining hepatic lipid homeostasis and preventing disease progression.

## 4. Pathological Consequences of Lipophagy Dysregulation

### 4.1. Amplification of Oxidative Stress

**Figure 3:**
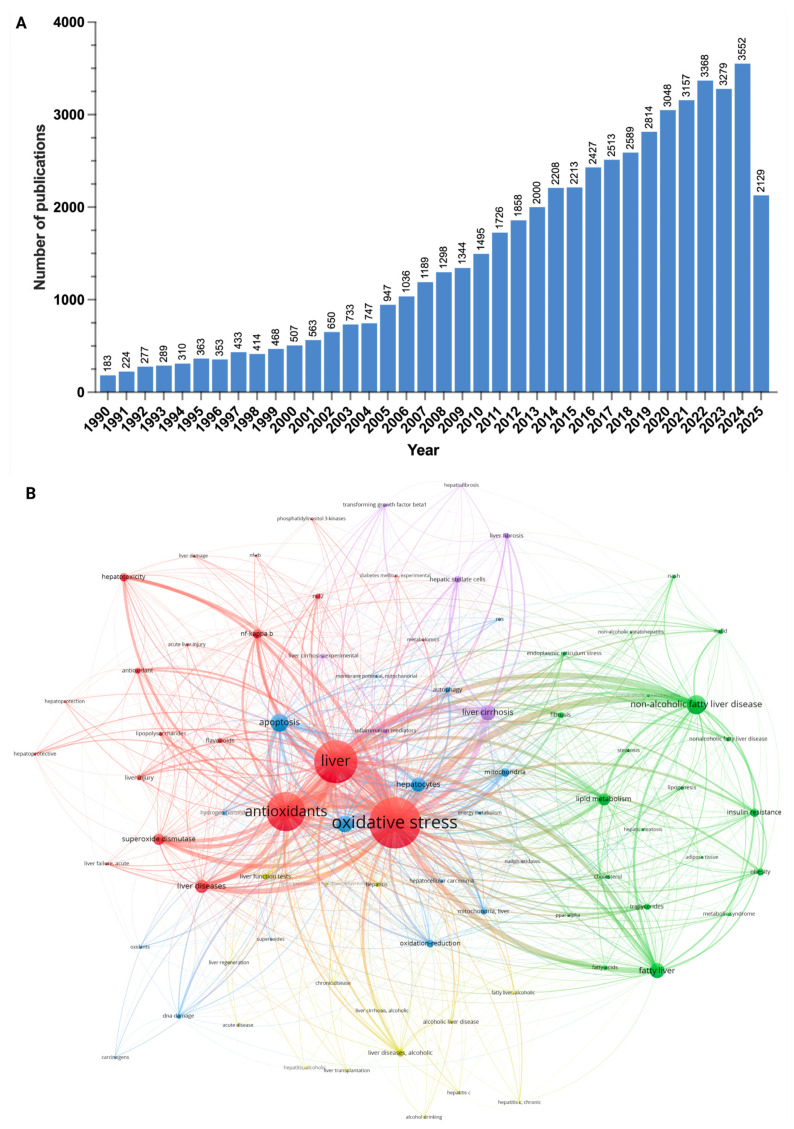
Visualization of a bibliometric analysis examining the relationship between oxidative stress and liver disease. (**A**) Annual number of English-language publications from 1990 to 2025. (**B**) Network map of co-occurring keywords, highlighting major research themes. Bibliographic data retrieved from the PubMed database on 8 July 2025.

Oxidative stress, resulting from an imbalance between the production of ROSs and the cellular antioxidant capacity, is a crucial factor in the progression of MASH [97,98,99]. Reflecting the growing focus on this pathological mechanism, research into the link between oxidative stress and liver disease has significantly increased over the past thirty years (Figure 3A). Further emphasizing this focus, a bibliometric analysis of co-occurring keywords indicates that “oxidative stress” serves as a central concept connecting two major thematic clusters in the MASLD research field (Figure 3B). The first cluster (red) involves mechanisms of cellular damage, such as “hepatotoxicity” and “NF-kappaB,” while the second cluster (green) focuses on the metabolic aspects of MASLD, including “lipid metabolism,” “insulin resistance,” and “fatty liver.” This analysis illustrates how the interplay between oxidative damage and liver pathophysiology has become a central hypothesis in contemporary liver research.

Recent evidence identifies lipophagy dysfunction as a key contributor to oxidative stress in MASLD. Its impairment fosters a lipotoxic environment that undermines critical cellular metabolic organelles, such as the mitochondria and endoplasmic reticulum. This dysfunction initiates a self-perpetuating cycle where the initial inability to clear lipids impairs the organelles essential for metabolic health, resulting in a marked increase in ROS production.

The most immediate consequence of impaired lipophagy is mitochondrial dysfunction, which serves as a primary source of ROSs in MASH [100]. When lipophagy is impaired, hepatocytes accumulate LDs and increasingly depend on cytosolic lipolysis to break down stored fats [101,102,103,104,105]. However, this compensatory pathway overloads mitochondria with FFAs beyond their β-oxidation capacity, leading to incomplete oxidation and electron leakage from the electron transport chain (ETC), elevating superoxide production [106]. Additionally, the buildup of toxic lipid species due to compromised lipophagy directly damages mitochondrial membranes and proteins, further disrupting ETC function and resulting in lipotoxicity and ROS generation [107,108]. Moreover, mitophagy, the selective removal of damaged mitochondria through autophagy, is often impaired alongside lipophagy in MASH, causing a buildup of dysfunctional, ROS-producing mitochondria and perpetuating a cycle of cellular damage [109,110].

**Figure 4:**
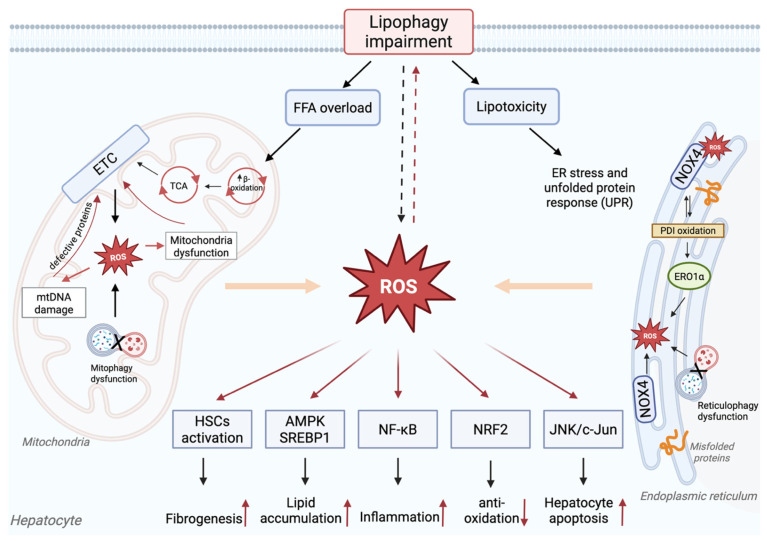
Lipophagy impairment plays a central role in the pathology of MASLD. In hepatocytes, dysfunctional lipophagy leads to an overload of FFAs and lipotoxicity, activating various ROS production pathways. The excess FFAs overwhelm mitochondrial β-oxidation and the tricarboxylic acid (TCA) cycle, causing dysfunction in the electron transport chain (ETC) and ROS leakage. This ROS damages mitochondrial DNA (mtDNA) and proteins, a situation worsened by impaired mitophagy, which hinders the removal of damaged, ROS-generating mitochondria. Simultaneously, lipotoxicity induces ER stress and the unfolded protein response (UPR). This event triggers a ROS-amplifying loop involving NOX4, PDI oxidation, and ERO1α, leading to increased ROS levels and accumulation of misfolded proteins. Impaired reticulophagy exacerbates this stress by failing to clear the damaged ER. The resultant severe oxidative stress activates multiple downstream signaling pathways that drive key features of MASH. These include the activation of hepatic stellate cells (HSCs) leading to fibrogenesis, modulation of AMPK/SREBP1 signaling to enhance lipid accumulation, activation of NF-κB to promote inflammation, impairment of the NRF2 antioxidant response, and activation of the JNK/c-Jun pathway leading to hepatocyte apoptosis. The illustration was mainly supported by these references [101,107,113,116,131].

Lipotoxicity resulting from impaired lipophagy also triggers severe endoplasmic reticulum stress and the unfolded protein response (UPR), making the ER a central site for ROS production in MASH [111]. A primary mechanism involves the activity of specific ROS-generating enzymes implicated in ER stress, which are often elevated in MASH [111,112]. Notably, the activity of NADPH Oxidase 4 (NOX4) increases, initiating a powerful oxidative amplification loop [113,114]. NOX4 is activated by stimuli common in MASH, such as inflammatory cytokines and metabolic signals like FFAs or angiotensin II, making it a major contributor to overall ROS production [115,116,117]. NOX4-derived ROS directly oxidizes the chaperone Protein Disulfide Isomerase (PDI), which activates ER Oxidoreductin 1 (ERO1α) [118]. Activated ERO1α then generates a significant burst of ROS during protein folding, substantially increasing the ER oxidative burden and contributing to liver injury [119,120] (Figure 4). Additionally, the cytochrome P450 enzyme CYP2E1 is often induced under MASH conditions, and its metabolic activity significantly contributes to ROS generation [121,122]. This situation worsens when impaired reticulophagy, the autophagic clearance of ER components, fails to remove aggregated proteins and damaged ER segments, prolonging stress signals [123].

In addition, other enzymes throughout the cell also enhance oxidative stress production. Different NOX isoforms, such as NOX1 and NOX2 in immune cells, are activated by the pro-inflammatory MASH environment [113,114]. Other enzymes, like xanthine oxidase, further increase the oxidative burden in the stressed liver [124]. As ROS production rises, chronic oxidative stress compromises and overwhelms cellular antioxidant defense systems [125,126,127]. The nuclear factor erythroid 2-related factor 2 (NRF2) pathway, a key regulator of antioxidant gene expression, often becomes dysfunctional or undergoes complex regulation in advanced disease stages [128]. Since autophagy supports antioxidant defense by removing oxidized proteins and organelles and regulating NRF2 activity, impaired lipophagy indirectly diminishes the cell’s ability to manage oxidative stress [129,130].

In summary, the failure of lipophagy acts as a critical upstream event that drives fatty acid accumulation and lipotoxicity. This dysfunction promotes ROS production from various cellular sources (e.g., mitochondria, ER, NOX enzyme systems), while simultaneously impairing the removal of damaged organelles via failed mitophagy and reticulophagy and weakening the cellular intrinsic antioxidant defenses. As illustrated in Figure 4, this cascade creates a feedback loop of oxidative damage, inflammation, and hepatocellular dysfunction that accelerates MASH progression.

### 4.2. Promotion of Inflammation

The lipotoxicity and intense oxidative stress resulting from impaired lipophagy are powerful triggers for hepatic inflammation in MASH. This process is driven by activating specific cellular stress-sensing pathways, notably through redox-sensitive signaling cascades. The chronic increase in ROSs acts as a signaling molecule, activating stress-activated protein kinases (SAPKs), such as JNK/c-Jun and p38 MAPK, as well as transcription factors like nuclear factor-kappa B (NF-κB) and activator protein-1 (AP-1 [131,132,133]. Once activated, these transcription factors move to the nucleus and promote the expression of a wide array of pro-inflammatory genes. This cascade prompts hepatocytes to produce and release cytokines and chemokines, including TNF-α, IL-6, and IL-1β, establishing and sustaining a chronic inflammatory environment in the liver [91].

Moreover, cellular stressors accumulating due to lipophagy failure, such as ROS, toxic lipid species like ceramides, and cholesterol crystals, are identified as danger-associated molecular patterns (DAMPs). These DAMPs are detected by pattern recognition receptors (PRRs) on both hepatocytes and resident hepatic immune cells [134]. A critical outcome of this detection is the activation of inflammasome complexes, particularly the NOD-like receptor protein 3 (NLRP3) inflammasome. The NLRP3 inflammasome serves as a multi-protein platform that, once assembled, converts pro-caspase-1 into its active enzymatic form. Activated caspase-1 then cleaves the pro-inflammatory cytokines pro-IL-1β and pro-IL-18 into their mature, highly potent forms. These cytokines are key drivers of sterile inflammation within the liver, promoting a strong inflammatory response and inducing a form of lytic, pro-inflammatory cell death known as pyroptosis [135,136].

The cytokines and chemokines released by stressed hepatocytes orchestrate the recruitment and activation of a substantial hepatic immune infiltrate, including resident macrophages (Kupffer cells) and circulating monocytes, neutrophils, and lymphocytes [137]. Once in the liver, these immune cells are activated by the local inflammatory environment and further amplify the inflammatory cascade. They contribute directly to hepatocellular injury by producing ROS, reactive nitrogen species, and additional pro-inflammatory cytokines, creating a self-perpetuating cycle of inflammation and tissue damage [138].

### 4.3. Progression to Liver Fibrosis

Chronic inflammation and ongoing injury to liver cells, caused by the dysregulation of lipophagy, set up a pro-fibrogenic environment that progresses to liver fibrosis. The key players in this development are the hepatic stellate cells. In a healthy liver, these cells remain inactive, mainly storing vitamin A. However, in the presence of the inflammatory and lipotoxic signals associated with MASH, they undergo a significant transformation into myofibroblast-like cells [139]. These activated HSCs, marked by the expression of α-smooth muscle actin (α-SMA), are responsible for the excessive production and accumulation of extracellular matrix (ECM) proteins, primarily fibrillar collagens, leading to progressive liver scarring [140].

HSC activation is driven by multiple interconnected mechanisms linked to lipophagy failure. Oxidative stress due to impaired lipophagy can directly activate HSCs, as ROSs serve as second messengers in fibrogenic signaling pathways [141,142,143]. More significantly, HSCs are activated through potent paracrine signaling from surrounding damaged hepatocytes and immune cells [144]. Dying hepatocytes release DAMPs and apoptotic bodies, stimulating resident immune cells, like Kupffer cells, to release strong pro-fibrogenic mediators [145]. Among these, tumor growth factor-β (TGF-β) is the most potent pro-fibrogenic cytokine. It binds to receptors on HSCs, initiating the canonical Smad signaling pathway (Smad2/3), which promotes the transcription of collagen genes such as COL1A1 and COL1A2 [146]. Other significant mediators, like platelet-derived growth factor (PDGF), encourage HSC proliferation, while inflammatory cytokines like TNF-α and IL-1β enhance their activation and survival [146].

Once activated, HSCs produce ECM and disrupt the normal balance of ECM turnover, leading to its accumulation. They secrete tissue inhibitors of metalloproteinases (TIMPs), which inhibit the activity of matrix metalloproteinases (MMPs), the enzymes responsible for breaking down old ECM [147,148]. Increased ECM production and decreased degradation are central to fibrosis progression. As lipophagy is compromised, lipotoxicity and oxidative stress rise, damaging hepatocytes. This damage triggers inflammation, activating HSCs and promoting fibrogenesis. Together, these events create a vicious cycle that drives the progression from steatohepatitis to advanced liver fibrosis, and ultimately cirrhosis.

## 5. The Dual Role of ATG7 in MASLD for Autophagy and Stress Response Regulation

The impairment of lipophagy in MASLD underscores the critical role of its fundamental molecular machinery [149]. At the heart of this process, the E1-like activating enzyme ATG7 is vital for autophagosome formation. However, recent research has uncovered that ATG7 also possesses autophagy-independent functions, particularly in regulating broader stress responses. These non-autophagic roles may be especially relevant in MASLD, where hepatocytes face chronic metabolic and oxidative stress.

### 5.1. The Multifaceted Roles of ATG7 Beyond Autophagosome Formation

ATG7 plays a crucial role in canonical autophagy, where its E1-like enzymatic activity is essential for the ATG12-ATG5 and LC3-PE conjugation systems [36,150]. Beyond this, ATG7 actively enhances cellular resilience by serving as a signaling and stress-sensing hub that regulates metabolic homeostasis and supports cell survival during intense stress periods [151,152]. Notably, ATG7 has a non-canonical function involving interaction with the tumor suppressor p53 [153]. Under metabolic stress, ATG7 can bind to p53 in both the cytoplasm and nucleus, directly influencing its regulation of target genes like CDKN1A (p21). This interaction allows ATG7 to control cell cycle progression and survival independently of its enzymatic activity. Additionally, ATG7 affects cell fate and cell cycle progression in specific contexts, such as promoting neuronal differentiation through its interaction with MDM2, a key regulator of p53 stability and activity [154]. These findings highlight the broader role of ATG7 in developmental and stress response pathways.

ATG7 also plays a direct role in metabolic sensing and adaptation. It functions as a metabolic sensor, responding to intracellular ATP levels and modulating the AKT1–PDCD4 signaling axis, a crucial pathway for regulating protein translation and promoting cell survival during metabolic stress [155]. Furthermore, ATG7 is involved in the cellular stress response machinery, including the Integrated Stress Response (ISR). In the liver, ATG7 is necessary for the full transcriptional activation of key ISR target genes, such as *GDF15* and *FGF21*, which have systemic effects on energy metabolism in response to amino acid starvation [156]. These findings reveal that ATG7 is not merely a component of the autophagic degradation machinery, but a multifaceted protein deeply involved in critical aspects of cellular signaling, stress adaptation, and metabolic regulation, often independent of its role in autophagosome formation.

### 5.2. The ATG7 Paradox in MASLD: Tissue-Specific Functions and Therapeutic Implications

ATG7 is pivotal in protecting hepatocytes from liver metabolic stress by facilitating lipophagic clearance of lipid droplets and preventing lipotoxic byproduct accumulation [157]. Its involvement in broader stress-adaptive responses, including p53 regulation, energy-sensing, and transcriptional reprogramming, forms a complex network for managing metabolic and oxidative stress in the context of metabolic dysfunction-associated steatotic liver disease [153]. Interestingly, the role of ATG7 in adipose tissue contrasts sharply with its protective function in the liver. Studies have shown that adipose-specific *Atg7* knockout in mice protects against diet-induced obesity and MASLD [158,159]. The primary mechanism behind this protection is the modulation of adipose–liver communication. Knocking out ATG7 in adipose tissue significantly reduces liver fat accumulation by decreasing FFA levels, thus protecting the liver from FFA overload [158]. Moreover, ATG7 is crucial for normal adipose tissue development, and its deletion impairs adipogenesis, reducing white adipose tissue mass and increasing adipocyte apoptosis [158,159].

Therefore, targeting ATG7 systemically for MASLD therapy presents a significant challenge. While enhancing ATG7 activity in the liver may help restore lipophagy and reduce hepatocellular stress, inhibiting it in adipose tissue offers metabolic benefits. Consequently, systemic modulation of ATG7 could yield conflicting outcomes depending on the organ targeted. This complexity presents a critical challenge for future therapies, necessitating strategies such as organ-specific drug delivery or targeting downstream pathways to avoid unintended effects and safely modulate this complex system.

## 6. Therapeutic Strategies by Modulating Lipophagy

### 6.1. Lipophagy as a Therapeutic Target

Enhancing lipophagy, given its crucial role in breaking down excess lipid droplets, presents a promising therapeutic approach for managing MASLD/MASH [160]. Boosting lipophagic activity could provide several advantages, such as reducing liver fat accumulation, easing lipotoxic stress, and decreasing inflammation [161]. By effectively eliminating lipid stores, functional lipophagy reduces the substrates available for mitochondrial ROS production and removes lipid droplets that may contain harmful pro-oxidant species [162,163]. However, significant risks are associated with therapeutically modulating lipophagy, making it a complex solution. An important challenge is the potential to cause an uncontrolled increase in FFAs, which are inherently toxic to cells [164,165]. When lipid droplet breakdown exceeds the cellular ability to utilize FFAs, the resulting influx can overwhelm mitochondrial oxidation, disrupt endoplasmic reticulum homeostasis, and activate other ROS-generating systems, like NOX enzymes, leading to widespread redox imbalance [166]. This disturbance can damage mitochondrial integrity, as seen with acylcarnitine accumulation when autophagic activity is high but lipid droplet formation is limited [167]. Ironically, this increased lipotoxicity can exacerbate oxidative stress, trigger pro-inflammatory signaling, cause liver cell injury, and promote fibrogenesis, all of which are key features in the progression of MASLD [164,166].

Thus, effective therapeutic strategies must take a comprehensive systems-level approach. It is crucial to activate lipophagy and ensure that mechanisms are in place for the efficient utilization or detoxification of released FFAs, such as through mitochondrial and peroxisomal β-oxidation. Additionally, given the broad effects of autophagy, caution is required when using general inducers like mTOR inhibitors, as they might disrupt cellular balance in unintended tissues or organelles and cause unwanted redox imbalances [168]. Furthermore, assessing the effectiveness of a therapy involves more than just relying on static markers; it requires reliable methods to measure actual autophagic flux and a comprehensive evaluation of the overall intervention impact on cellular oxidative stress and liver cell health. Without careful management, strategies to restore lipophagy might inadvertently replicate or accelerate MASH-like pathology rather than resolve it.

### 6.2. Pharmacological Approaches in Lipophagy

Pharmacological approaches for addressing MASH, especially concerning lipophagy dysregulation and related oxidative stress, are increasingly concentrating on three interconnected goals, which are as follows: (1) reestablishing effective lipophagic flux to manage the burden of lipid droplets; (2) reducing oxidative stress, which both arises from and worsens lipophagy dysfunction; and (3) enhancing liver and overall metabolic health to support autophagic processes and improve redox equilibrium.

#### 6.2.1. Autophagy Activators for MASLD

A key strategy involves directly stimulating lipophagy to enhance the removal of harmful lipid droplets. This method targets fundamental signaling pathways to restore cellular balance and reduce lipotoxicity. As outlined in Table 1, this approach focuses primarily on inhibiting mTORC1, a central negative regulator of autophagy, or activating AMPK, a central positive regulator

Inhibiting mTORC1 is a direct way to boost autophagic activity. Rapamycin, a well-known mTORC1 inhibitor, has been shown to decrease liver fat accumulation, lower serum alanine aminotransferase (ALT) levels, and improve insulin sensitivity in mouse models of fatty liver disease [169,170]. Torin-1, another potent inhibitor of mTORC1 and mTORC2, increases autophagic activity and promotes TFEB-mediated lysosomal biogenesis. In models of chronic liver injury, Torin-1 has been linked to reduced liver triglyceride levels and improved liver function [176]. Genetic studies further support the therapeutic potential of this pathway. For example, hepatic overexpression of SNRK or deletion of FLCN suppresses mTORC1 activity, enhancing autophagy and reducing liver fat, fibrosis, and inflammation [173,175].

On the other hand, AMPK is an appealing target for MASLD treatment, as it connects cellular energy status to the induction of autophagy and redox balance. Metformin, in addition to its antidiabetic effects, has been reported to protect mice with MASLD by reducing liver fat accumulation and improving insulin sensitivity through the regulation of the AMPK/mTOR pathway, as it activates AMPK and thereby inhibits mTORC1 [178,179]. Direct AMPK activators like PXL770 have shown promise in preclinical models by improving systemic metabolic health, reducing lipogenesis, and enhancing insulin sensitivity, all beneficial in MASLD [171,172]. Ezetimibe, known for inhibiting cholesterol absorption, activates AMPK and increases the expression of autophagy-related proteins, significantly reducing liver fat accumulation, inflammation, and fibrosis [174]. Liraglutide, a GLP-1 receptor agonist, similarly stimulates AMPK signaling, increasing p-AMPK, Beclin-1, and LC3-II/LC3-I, effectively reducing liver fat content and oxidative stress [177]. Some compounds, like Buddleoside, achieve enhanced efficacy by activating AMPK and inhibiting mTORC1, significantly decreasing liver fat, inflammation, and fibrosis [181].

These findings illustrate that modulating the core autophagy signaling network is a validated and promising strategy for MASLD. This approach can be achieved through direct, targeted activators, or the diverse effects of existing metabolic drugs. Together, these approaches underscore the therapeutic potential of restoring lipophagic activity to manage lipid clearance and mitigate the downstream pathology of MASH.

#### 6.2.2. Mechanisms of AMPK/mTORC1 Regulation

The AMPK/mTORC1 signaling axis is crucial for cellular metabolic regulation, making it a prime target for therapeutic interventions in MASLD. Treatments targeting this pathway operate through direct or indirect mechanisms, offering distinct advantages for managing the disease.

A primary strategy involves activating AMPK to inhibit anabolic processes such as lipogenesis, and to stimulate catabolic processes like lipophagy and fatty acid oxidation [182,183]. Various pharmacological agents can activate AMPK via different routes, altering cellular energy states. For instance, metformin inhibits mitochondrial complex I, reducing ATP production and increasing the AMP/ATP ratio, which allosterically activates AMPK [184]. Similarly, Liraglutide and GLP-1 receptor agonists activate AMPK indirectly through a receptor-mediated signaling cascade that involves protein kinase A (PKA) phosphorylation [177]. This links systemic hormonal signals to the cellular energy-sensing network. Unlike these indirect methods, PXL770 is a pioneering direct AMPK activator that binds to an allosteric site on the AMPK complex, causing a conformational change that activates the enzyme, even in nutrient-rich conditions [185]. Natural compounds like quercetin and berberine may also exert effects through direct interactions and indirect mitochondrial function modulation [186,187].

Additionally, inhibiting mTORC1 is another strategy to enhance autophagy and lipophagy. Rapamycin and its analogs (rapalogs) bind to the FKBP12 protein, forming a complex that attaches to the FRB domain of mTOR [188]. This event prevents mTORC1 from accessing its substrates without blocking the kinase-active site [189]. Alternatively, ATP-competitive kinase inhibitors like Torin-1 directly target the mTOR catalytic site, affecting both mTORC1 and mTORC2 activities [190]. While this dual inhibition can lead to broader and more potent effects, it also increases the risk of off-target effects by impacting mTORC2-dependent pathways. This diversity in pharmacological approaches emphasizes the importance of the specific mechanism of action, which determines the therapeutic efficacy, target specificity, and safety of drugs for treating MASLD.

#### 6.2.3. Integrating Autophagy Modulation with Antioxidant Effects

An advanced therapeutic approach for MASH involves using compounds that enhance autophagy and provide direct antioxidant or anti-inflammatory effects. This strategy tackles the pathogenic cycle where excess lipid accumulation leads to oxidative stress, which subsequently hampers autophagic function. By boosting cellular cleanup through lipophagy and strengthening antioxidant defenses, dual-action agents offer a more comprehensive and potentially synergistic therapeutic advantage (see Table 2).

Many agents that activate the AMPK pathway also have these dual properties. For instance, the flavonoid quercetin induces autophagy via AMPK activation and delivers direct antioxidant and anti-inflammatory effects, reducing lipid accumulation and fibrosis. Similarly, Liraglutide, a GLP-1 receptor agonist, activates AMPK, enhances lipophagy, and reduces hepatic ROS accumulation. Its coordinated effects on lipid metabolism and oxidative balance underscore its potential as a dual-action therapy for MASLD. Bergamot Polyphenol Fraction offers another example, where its ability to induce autophagy is complemented by a reduction in both hepatic lipid accumulation and its associated oxidative stress [193,194,195].

In addition to leveraging AMPK, another key strategy for dual-action therapy is targeting the cellular antioxidant machinery, primarily through the NRF2 pathway [203]. In MASH, NRF2 activity is often compromised due to disruptions in the autophagic degradation of its inhibitor, KEAP1. Agents that activate NRF2 can restore redox balance while indirectly supporting autophagy [128]. Quercetin, for example, exhibits these multifaceted effects by activating AMPK while inhibiting mTORC1, significantly reducing hepatic lipid stores, inflammation, and oxidative stress, along with ameliorating ALT and AST levels [187,192].

Overall, dual-action agents that combine autophagy enhancement with antioxidant support offer a comprehensive strategy to break the pathological cycle of steatosis, oxidative injury, and inflammation. By addressing both lipid overload and redox imbalance, these compounds hold significant promise for disrupting the pathological progression of MASH.

#### 6.2.4. Therapeutic Considerations

The development of effective therapies for MASH must address the complex challenges previously discussed, including the dual nature of autophagy activation and the cycle linking oxidative stress to autophagic dysfunction [204,205,206,207]. Therefore, the future of MASH pharmacology likely rests with multi-target agents that can restore cellular balance on multiple levels. The most promising strategies will be those that can simultaneously restore functional autophagic flux to safely clear lipids and damaged organelles while enhancing antioxidant defenses through mechanisms like NRF2 activation to manage the existing oxidative burden. Moreover, these agents must improve overall metabolic health, such as insulin sensitivity, to address the primary causes of hepatic lipid accumulation.

While these drugs are effective in treating MASLD/MASH, they may also lead to potential side effects. Metformin is typically well-tolerated, but it can cause gastrointestinal issues and has a rare risk of lactic acidosis, particularly in patients with severe renal impairment [208,209]. Long-term use of metformin also requires monitoring for vitamin B12 deficiency [209]. On the other hand, broad mTOR inhibitors like rapamycin can cause significant immunosuppression and may result in paradoxical metabolic dysregulation due to mTORC2 inhibition [210]. Other medications have their challenges; for example, liraglutide may increase the risk of pancreatitis and gallbladder disease [211,212], while ezetimibe has a genetically predicted risk of gallstones [213]. Natural compounds also bring specific considerations. Quercetin’s effectiveness is limited by poor oral bioavailability [214]. Bergamot may interact with other medications by inhibiting CYP3A4 enzymes [215], and even the generally safe melatonin should be noted for its primary effect of inducing drowsiness [216,217]. This evidence highlights the importance of considering a drug’s safety and tolerability as crucial to its clinical success, alongside its intended mechanism of action.

Successfully implementing these sophisticated strategies in clinical practice will require significant advancements. Reliable biomarkers are needed to accurately measure autophagic flux in patients, moving beyond static markers. Additionally, a deeper understanding of patient-specific factors that influence the balance between lipophagy, lipotoxicity, and redox signaling will be crucial for personalizing treatment and fully realizing the therapeutic potential of these next-generation therapies.

## 7. Strengths, Limitations, and Future Directions: A SWOT Analysis

This review examines the potential of targeting lipophagy for MASLD through a SWOT analysis. This framework aids in understanding the therapeutic promise of modulating lipophagy while identifying existing challenges and future research directions.

### 7.1. Strength: Targeting a Core Pathological Driver

Lipophagy is crucial for maintaining liver lipid balance, and its dysfunction is a significant factor in MASLD development and progression. This review highlights that impaired lipophagic activity results in excessive lipid accumulation, lipotoxicity, and oxidative stress, contributing to ongoing liver cell damage, inflammation, and fibrosis. The strength of targeting lipophagy lies in its ability to tackle a fundamental pathological process in MASLD, presenting a promising therapeutic strategy with potentially wide-ranging benefits.

### 7.2. Limitations (Weaknesses): Therapeutic Risks and Measurement Challenges

Despite its potential, several key challenges must be addressed to safely and effectively target lipophagy in clinical settings. Therapeutically enhancing lipophagy must be approached cautiously due to the risk of inducing lipotoxicity and mitochondrial dysfunction by overloading cellular metabolism [164,165]. Additionally, accurately measuring autophagic activity remains a significant challenge, as current static markers like LC3-II fail to capture functional activity adequately [218,219,220]. The limitations of current therapeutic tools further exacerbate this gap in clinical translation. Broad-spectrum modulators such as mTOR inhibitors suffer from significant off-target effects and the lack of liver-specific drug delivery, complicating their clinical utility, especially given patient variability in metabolic and autophagic responses [221]. Furthermore, the absence of established trial endpoints to evaluate lipophagic restoration in clinical trials poses an additional hurdle for new therapeutic approaches.

### 7.3. Opportunities: Precision Medicine and Rational Drug Design

The limitations, as mentioned earlier, highlight key opportunities for future research and drug development. To mitigate adverse effects, new strategies must ensure a balanced coordination of lipid mobilization, with downstream pathways like mitochondrial oxidation or lipid re-esterification [164]. Identifying patients with primary lipophagy impairment will be essential for personalizing treatments. Additionally, overcoming the limitations of broad-spectrum autophagy modulators necessitates a shift towards rational drug design. This involves developing liver-specific delivery systems, such as nanomedicine, and creating multi-target agents that can restore lipophagy while enhancing antioxidant defenses and metabolic resilience [222,223,224]. Collectively, these strategies could enhance the efficacy and safety of therapies based on lipophagy modulation.

### 7.4. Threats: Underlying Biological Complexities

The potential of targeting lipophagy therapeutically may be compromised by the underlying biological complexities of the system. Gaining a deeper understanding of the relationship between oxidative stress and autophagy in MASLD is essential. For instance, certain ROS sources can directly impair autophagic proteins like ATG3 and ATG7, worsening cellular dysfunction. Chronic autophagy suppression disrupts redox balance and accelerates liver damage [8,84,112,225]. Adding to this complexity, the emerging evidence of autophagy-independent roles of key proteins like ATG7 introduces another layer of complexity that could affect therapeutic outcomes, making it crucial to explore these functions as an essential yet underexplored research area [150,154,226,227].

## 8. Methods

### 8.1. Literature Search Strategy

This narrative review was developed through a multi-stage process, involving an extensive literature search, abstract and full-text article screening, and synthesis of the findings. Key scientific databases, such as PubMed/MEDLINE, Scopus, Web of Science, and Google Scholar, were queried to identify pertinent studies. The final literature search was conducted in July 2025, focusing on English-language publications, including peer-reviewed articles and authoritative book chapters. The primary keyword “lipophagy” was used alongside secondary terms like “MASLD,” “MASH,” “oxidative stress,” “hepatic steatosis,” “lipotoxicity,” and “metabolic dysfunction.” Additionally, targeted searches combined these terms with specific molecular pathways (e.g., “autophagy,” “NRF2,” “mitophagy,” “ATG7”) to obtain detailed mechanistic insights. After the database search, all retrieved titles and abstracts were assessed for relevance to the topic. The main eligibility criterion was that the study should analyze the role or mechanism of lipophagy or autophagy in the context of liver lipid metabolism and MASLD/MASH pathophysiology. The information from the selected articles was synthesized to create the narrative review presented here.

### 8.2. Bibliometric Analysis

To conduct a bibliometric analysis of publications related to “oxidative stress” in the context of “liver disease,” we retrieved data from PubMed, an accessible biomedical and life sciences literature database. The search focused on literature published between 1995 and 2025, filtering for English-language original research and review articles on oxidative stress in liver disease, while excluding preprints. The detailed search query was structured as follows: “(liver disease[MeSH Terms] OR liver disease OR hepatic disease OR liver cirrhosis) AND (antioxidants[MeSH Terms] OR antioxidants OR antioxidative Stress)”.

The trend of annual publications was plotted using GraphPad Prism (v.10.5.0). For the network analysis, the bibliographic data were imported into VOSviewer software (v.1.6.20). A co-occurrence map of keywords was generated using the “All Keywords” field and the full counting method, with a minimum occurrence threshold of 40 for each keyword. Out of 15,186 total keywords identified in the dataset, 440 met this threshold. These 440 keywords were then manually reviewed for relevance to ensure thematic accuracy of the research clusters before generating the final visualization.

## 9. Conclusions

Lipophagy plays a vital role in maintaining hepatic lipid balance, and its dysfunction is increasingly associated with the progression of MASLD. This review highlights that impaired lipophagic flux results in lipid accumulation, lipotoxicity, and oxidative stress, creating a cycle of liver cell injury, inflammation, and fibrosis. Therefore, lipophagy represents a significant pathological mechanism and a critical therapeutic target in the management of MASLD.

While the potential is promising, clinically targeting this process poses substantial challenges. As discussed, the primary challenge lies in transitioning from broad modulation to precise and balanced interventions. Future advancements will depend on developing therapeutic strategies tailored to patient-specific defects, ensuring the safe coordination of lipophagic flux restoration with the cell’s metabolic capacity.

In conclusion, a comprehensive understanding of the mechanisms underlying lipophagy presents a significant opportunity for improving the management of MASLD. Enhancing this understanding will pave the way for novel biomarkers of disease progression and therapeutic strategies that restore cellular balance, offering a clear path to prevent the progression of liver disease.

## Figures and Tables

**Table 1:** Therapeutic agents for MASLD exhibiting direct autophagy-activating effects.

Compounds or Target Genes	Regulation of Autophagy-Associated Proteins	Effects on MASLD	Reference
Rapamycin	↓ mTORC1, ↑ LC3-II/LC3-I	↓ Lipid accumulation, ↓ ALT, ↑ Insulin sensitivity	[169,170]
PXL770	↑ p-AMPK	↓ Lipogenesis, ↑ Insulin sensitivity, ↓ Circulating lipid	[171,172]
*SNRK* overexpression	↓ mTORC1, ↑ p62, ↑ LC3-II/LC3-I, ↑ ATG7	↓ Lipid accumulation, ↑ Fatty acid oxidation	[173]
Ezetimibe	↑ p-AMPK, ↑ LC3-II/LC3-I, ↑ ATG7, ↑ ATG7, ↑ ATG12, ↑ ULK1, ↑ BECN1, ↑ p62	↓ Lipid accumulation, ↓ Inflammation, ↓ Fibrosis	[174]
*FLCN* deletion	↓ mTORC1, ↑ TFE3, ↑ Lysosomal biogenesis	↓ Lipid accumulation, ↓ Fibrosis, ↓ Inflammation	[175]
Torin-1	↓ mTORC1, ↑ LC3-II/LC3-I, ↑ TFEB	↓ Lipid accumulation, ↓ ALT	[176]
Liraglutide	↑ p-AMPK, ↑ LC3-II/LC3-I, ↑ Beclin-1	↓ Lipid accumulation, ↓ Liver injury, ↓ Oxidative stress	[177]
Metformin	↑ p-AMPK, ↓ mTORC1, ↑ LC3-II/LC3-I, ↑ TFEB	↓ Lipid accumulation, ↑ Insulin sensitivity	[178,179]
Stevioside	↑ LC3-II/LC3-I, ↑ p-AMPK	↓ Lipid accumulation	[180]
Buddleoside	↑ p-AMPK, ↓ mTORC1, ↑ LC3-II/LC3-I, ↑ TFEB,	↓ Lipid accumulation, ↑ Insulin sensitivity, ↓ Inflammation, ↓ Hepatic Fibrosis	[181]

**Table 2:** Therapeutic agents for MASLD exhibiting both autophagy-enhancing and antioxidant effects.

Compounds	Oxidative Markers	Effect on Autophagy	Effects on MASLD	References
Ergothioneine	↑ CAT, ↑ SOD3, ↑ GPX4, ↑ GSH	↑ ATG5, ↑ Beclin-1, ↓ p62	↓ Inflammation, ↓ Fibrosis, ↓ Lipid accumulation,	[191]
Berberine	↑ SOD, ↑ CAT, ↑ GPX	↑ p-AMPK, ↓mTORC1, ↑ LC3-II/LC3-I, ↑ Beclin-1	↓ Liver injury, ↓ Lipid accumulation, ↓ Oxidative stress, ↓ ALT, ↓ AST	[186]
Quercetin	↓ ROS	↑ p-AMPK, ↑ ATG5, ↑ ATG12, ↑ LC3-II/LC3-I, ↑ PINK1, ↑ Parkin,	↓ ALT, ↓ AST, ↓ Lipid accumulation, ↓ Inflammation, ↓ Oxidative stress	[187,192]
Bergamot Polyphenol Fraction	↓ ROS	↑ LC3-II	↓ Lipid accumulation, ↓ Liver inflammation	[193,194,195]
Sesamin	↓ ROS, ↑ GSH, ↓ MDA	↑ LC3-II	↓ Lipid accumulation, ↓ Oxidative stress	[196]
2,3,5,4′-Tetrahydroxystilbene-2-O-β-D-glucoside (TSG)	↓ ROS, ↑ SOD	↑ LC3-II, ↑ p-AMPK	↓ Lipid accumulation,	[197]
Docosahexaenoic acid (DHA)	↓ ROS, ↑ SOD	↑ LC3-II, ↓ p62	↓ Oxidative stress, ↓ ALT, ↓ AST	[198]
5-O-Demethylnobiletin (5-DN)	↓ ROS, ↑ SOD	↑ LC3-II/LC3-I	↓ Lipid accumulation, ↓ Inflammation, ↓ Fibrosis ↓ Oxidative stress,	[199]
Ellagic Acid (EA)	↓ ROS, ↑ SOD	↑ LC3-II, ↑ ATG5, ↓ BECN1	↓ Lipid accumulation, ↓ Inflammation, ↓ Fibrosis ↓ Oxidative stress,	[200]
Melatonin	↓ ROS, ↑ SOD	↑ LC3-II, ↓ p62	↓ Lipid accumulation, ↓ Oxidative stress	[201]
Micronized Palmitoylethanolamide (m-PEA)	↓ ROS, ↑ SOD	↑ p-AMPK, ↑ LC3-II/LC3-I	↓ Lipid accumulation, ↓ Inflammation, ↓ Fibrosis ↓ Oxidative stress,	[202]

## Data Availability

Not applicable.

## Abbreviations

ALT: alanine aminotransferase; AMPK: AMP-activated protein kinase; ANXA2: Annexin A2; AST: aspartate aminotransferase; ATG: autophagy-related genes; ATGL: adipose triglyceride lipase; CMA: chaperone-mediated autophagy; DAMP: danger-associated molecular pattern; ER: endoplasmic reticulum; ERO1a: ER oxidoreductin 1 alpha; ESCRT: endosomal sorting complexes required for transport; ETC: electron transport chain; FFA: free fatty acid; HSC: hepatic stellate cell; HSL: hormone-sensitive lipase; LAL: lysosomal acid lipase; LC3: Microtubule-associated protein light chain 3; LD: lipid droplet; LIR: LC3-interacting region; MASH: metabolic dysfunction-associated steatohepatitis; MASLD: metabolic dysfunction-associated steatotic liver disease; MMPs: metalloproteinases; mTORC: mammalian target of rapamycin complex; NF-kB: nuclear transcription factor kappa B; NLRP3: NOD-like receptor protein 3; NOX4: NADPH oxidase 4; NRF2: nuclear factor erythroid-related factor 2; PDGF: platelet derived growth factor; PE: phosphatidylenthanolamine; PKA: protein kinase A; PLIN: Perilipin; ROS: reactive oxygen species; SAPK: stress-activated protein kinase; a-SMA: alpha-smooth muscle actin; TAG: triacylglycerol; TCA: tricarboxylic acid; TLR: toll-like receptor; TGF-b: tumor growth factor beta; TNF-a: tumor necrosis factor alpha; UPR: unfolded protein response; VLDL: very low-density lipoprotein.
